# Trends in age at male circumcision and its determinants in Rwanda

**DOI:** 10.1186/s12981-025-00836-3

**Published:** 2026-01-09

**Authors:** Theogene Kubahoniyesu, Riziki Kagabo, Emmanuel Ngendahimana, Hassan Mugabo, Jean Paul Nsengiyumva, Florence Namalinzi

**Affiliations:** 1 African Centre of Excellence in Data Science, University of Rwanda, Kigali, Rwanda; 2 Optima Data Solutions Limited, Kigali, Rwanda; 3 Research Innovation and Data Science, Rwanda Biomedical Centre, Kigali, Rwanda; 4 Department of Health Science, Kibogora Polytechnic, Nyamasheke, Rwanda; 5 Makerere University-Johns Hopkins University Research Collaboration (MU-JHU), Kampala, Uganda

**Keywords:** Male circumcision, Prevalence, Survival analysis, Rwanda

## Abstract

**Background:**

Voluntary medical male circumcision (VMMC) is a well-established public health intervention proven to reduce the risk of human immunodeficiency virus (HIV) infection. Its protective benefit is greatest when performed early, ideally within the first few days after birth or before sexual debut. Over the past decade, Rwanda has made remarkable progress in scaling up circumcision services as part of its comprehensive HIV prevention strategy. Despite these advances, there remains limited evidence on the timing of circumcision and the factors that influence when men choose to undergo the procedure. Understanding these determinants is essential for optimizing the preventive effectiveness of VMMC and improving service uptake across different age groups.

**Methods:**

This study employed a retrospective cross-sectional design using data from the Rwanda Demographic and Health Surveys (RDHS) conducted in 2010, 2015, and 2020. The analysis included 15,965 men aged 15–59 years. Kaplan–Meier survival curves and log-rank tests were used to examine differences in the timing of circumcision across population subgroups, while Cox proportional hazards regression models were applied to identify factors associated with earlier circumcision. All analyses incorporated sampling weights and accounted for the complex survey design of the RDHS to ensure nationally representative estimates.

**Results:**

The prevalence of male circumcision among Rwandan men increased from 13.3% in 2010 to 30.6% in 2015 and further to 52.4% in 2020. The median age at circumcision was 15 years (95% CI: 14–16) in 2010, increased to 17 years (95% CI: 17–18) in 2015, and declined to 16 years (95% CI: 16–16) in 2020. In 2020, men with higher education had a 30% higher hazard of circumcision compared with those with no formal education (AHR = 1.30; 95% CI: 1.04–1.63; *p* = 0.020), indicating that circumcision occurred earlier among more educated men. Similarly, watching television frequently was associated with a 21% higher hazard of circumcision (AHR = 1.21; 95% CI: 1.10–1.33; *p* < 0.001), suggesting that media exposure accelerated uptake. In contrast, older men were slower to undergo circumcision compared with those aged 15–19 years, with hazards decreasing among those aged 20–24 years (AHR = 0.41; 95% CI: 0.36–0.46; *p* < 0.001) and 25–29 years (AHR = 0.24; 95% CI: 0.20–0.28; *p* < 0.001). Likewise, men residing in rural areas had a 21% lower hazard of circumcision relative to their urban counterparts (AHR = 0.79; 95% CI: 0.72–0.85; *p* < 0.001), indicating delayed uptake in rural settings.

**Conclusions:**

Male circumcision uptake in Rwanda has increased markedly over the past decade, with the most significant gains observed among younger men. Sustained efforts that strengthen health education, expand media-based awareness campaigns, and implement targeted approaches for older and rural populations could further promote earlier circumcision uptake and enhance the effectiveness of national HIV prevention programs.

## Introduction

Male circumcision is one of the oldest surgical practices worldwide, involving the removal of the foreskin of the penis [1]. It offers important health, cultural, and religious benefits. In public health, male circumcision has been endorsed by the World Health Organization (WHO) and Joint United Nations Programme on HIV/AIDS (UNAIDS) as a critical HIV prevention strategy in high-burden regions, with evidence showing it reduces heterosexual HIV transmission risk by approximately 60% [2]. Globally, around 37–39% of males are circumcised, with prevalence highest in regions dominated by Muslim and Jewish populations [3]. In areas where circumcision is not traditionally practiced, the decision is often influenced by public health campaigns and individual choice, reflecting its growing role as a preventive health measure [4]. 

Sub-Saharan Africa, which bears the heaviest burden of HIV infection globally (67%), has been a major focus of efforts to expand voluntary medical male circumcision (VMMC) [5, 6]. Several countries across sub-Saharan Africa, including South Africa, Kenya, Zimbabwe, Uganda, and Tanzania, have reported significant increases in circumcision coverage following intensive national campaigns [7]. Estimates show that circumcision prevalence across sub-Saharan Africa ranges widely from 20% to 80%, depending on the country and cultural context [8]. However, despite overall progress, substantial disparities persist, particularly in rural and underserved communities where access to health services remains limited [8]. 

In East Africa, circumcision coverage varies across countries due to differences in baseline prevalence, population size, and the cultural or religious practice of traditional circumcision. The Democratic Republic of Congo, where circumcision is deeply rooted in cultural and religious traditions, has the highest prevalence at approximately 97% [3]. Kenya follows with 91% through large-scale voluntary medical male circumcision campaigns, and Tanzania with 80%, reflecting both traditional and medical circumcision practices [9], while Burundi reports 61.7% [10]. Rwanda where circumcision is primarily medical rather than traditional remains comparatively lower at 56% among men aged 15–49 years [11]. Lastly, Uganda’s coverage nearly doubled from 26% in 2011 to 43% in 2016 and remains the lowest in East Africa [12]. These contextual differences highlight the need to consider cultural norms, initial coverage levels, and demographic variations when designing and evaluating circumcision programs across the region.

In Rwanda, male circumcision has primarily been promoted as part of the national HIV prevention strategy, with VMMC being the most common approach. Early infant circumcision is being practiced in some health facilities [13]. Traditional circumcision is rare and not culturally widespread, though isolated non-medical or partial circumcisions may occur and could influence self-reported status. National campaigns have mainly targeted adolescents and young adults, leading to increased coverage but with persistent disparities among rural and low-income men [14]. Limited evidence, however, exists on the timing of circumcision, which is crucial for maximizing its preventive impact and service utilization rate.

This study employs survival analysis methods to examine not only the prevalence but also the timing of male circumcision in Rwanda. Survival analysis is well-suited for analysing time-to-event data, allowing for a precise evaluation of age-specific trends and factors influencing earlier circumcision. Understanding the timing is critical because circumcising males before sexual debut maximizes the protective benefit against HIV acquisition [15]. According to the Rwanda Demographic and Health Survey (RDHS 2020), the median age at first sexual intercourse among men aged 25–59 years is 22.2 years, suggesting that circumcision occurring during adolescence or earlier would provide the greatest preventive advantage [11]. By identifying factors associated with earlier or delayed circumcision, the study aims to inform policies targeting specific age groups and reducing barriers to timely uptake, thereby enhancing public health outcomes in Rwanda and similar contexts.

## Methods and materials

### Study design and period

This study employed a retrospective cross-sectional design using data from three rounds of the Rwanda Demographic and Health Survey (RDHS) conducted in 2010, 2015, and 2020. The objective was to assess the trends in age at male circumcision and identify factors associated with early circumcision over time.

### Study setting

The study was conducted in Rwanda, a landlocked country in East Africa with a population of over 13,2 million [16]. The RDHS collects nationally representative data on a wide range of socio-demographic and health indicators, including male circumcision, reproductive health, and HIV-related behaviors.

### Source of data

The data used in this study were extracted from the men’s datasets (MR files) of the RDHS 2010, 2015, and 2020. These surveys were conducted by the National Institute of Statistics of Rwanda (NISR) in collaboration with the Rwanda Ministry of Health and ICF International, with funding from various partners.

### Sampling methods

The RDHS employed a two-stage stratified cluster sampling method. In the first stage, enumeration areas (EAs) were selected from the national sampling frame. In the second stage, a fixed number of households were randomly selected from each EA. All eligible men aged 15–59 residing in the selected households were interviewed, interviews were conducted primarily in Kinyarwanda, the national language of Rwanda, with questionnaires translated from English to ensure clarity and cultural appropriateness. Sampling weights were applied to ensure representativeness at the national level.

### Description of variables

#### Dependent variables

**Age at circumcision**: This was the outcome variable of interest, measured in completed years. For survival analysis, it was treated as a time-to-event variable with circumcision coded as an event (1 = circumcised, 0 = not circumcised) and age as the time scale.

#### Early male circumcision (early MC)

In this study, early male circumcision (early MC) refers to circumcision performed during adolescence or before sexual debut.

#### The independent variables

They included respondents’ age group, marital status, highest level of education attained, place of residence, religious affiliation, media exposure such as listening to radio and watching television, household wealth index, age at first sexual intercourse, and whether the respondent had ever been tested for HIV or not, etc. These factors were analysed to assess their association with the timing of male circumcision.

### Statistical analysis

#### Data preprocessing

Data were cleaned, and variables were harmonized across the three datasets. Age at circumcision was used to create a time-to-event variable. Only male respondents aged 15–59 who reported circumcision status were included. Sampling weights were normalized and applied.

#### Descriptive analysis

Frequencies and percentages were used to describe socio-demographic characteristics by survey year. Trends in circumcision prevalence were illustrated using a line graph.

#### Survival analysis

- Kaplan-Meier curves and log-rank tests were used to compare age at circumcision across categories of independent variables.
- Cox proportional hazards regression models were used to estimate adjusted hazard ratios (AHRs) and 95% confidence intervals (CIs) for factors associated with earlier circumcision.
- Analyses were conducted separately for each survey year using R software (R 4.3.3), and significance was assessed at *p* < 0.05.

### Ethics statement

The study was based on publicly available, de-identified DHS datasets. Permission to use the data was obtained from the DHS Program upon request. Ethical approval for data collection was granted by the Rwanda National Ethics Committee and ICF’s Institutional Review Board. Since the analysis used secondary data, no additional ethical approval was required.

## Results

### Socio-demographic characteristics of survey participants

Across the three Rwanda Demographic Surveys from 2010 to 2020, the majority of respondents were aged 15–19 years, accounting for 23% in 2010, 21% in 2015, and 24% in 2020, while the proportion aged 25–29 years showed a gradual decline over time compared to other age groups. Regarding marital status, 53% of participants reported being married across the years, with single individuals representing around 45%. In terms of education, primary education was predominant, with 67% in 2010 decreasing to 61% in 2020, while secondary or higher education levels increased from 21% to 30%. Urban residence rose from 17% in 2010 to 26% in 2015 before slightly dropping to 23% in 2020.

Listening to the radio frequently declined from 88% to 80% over the period, while television viewership remained around 30%. Most respondents reported having their first sexual experience between the ages of 15 and 24 years, with a slight decrease from 54% in 2010 to 51% in 2020. HIV testing uptake increased sharply from 71% in 2010 to 81% in 2015 but later decreased to 66% in 2020. In terms of wealth distribution, individuals categorized as rich constituted the largest proportion, although their share declined slightly from 48% in 2010 to 45% in 2020 (Table 1).

**Table 1:** Socio-demographic characteristics of male participants in the Rwanda demographic and health Surveys, 2010–2020

Variables	2010		2015		2020	
	Frequency (*n* = 3,241)	Percent (%)	Frequency (*N* = 6,212)	Percent (%)	Frequency (*N* = 6,512)	Percent (%)
*Age Group (Years)*						
15–19	730	22.5	1,279	20.6	1,534	23.6
20–24	573	17.7	998	16.1	954	14.7
25–29	538	16.6	963	15.5	734	11.3
30–34	378	11.7	931	15.0	816	12.5
35–39	259	8.0	559	9.0	784	12.0
40–44	233	7.2	469	7.5	570	8.8
45–49	209	6.4	381	6.1	440	6.8
Above 50	321	9.9	632	10.2	680	10.4
*Marital status*						
Married	1,709	52.7	3,325	53.5	3,440	52.8
Separated	80	2.5	143	2.3	150	2.3
Single	1,452	44.8	2,744	44.2	2,922	44.9
*Education level*						
No formal education	402	12.4	658	10.6	582	8.9
Primary	2,165	66.8	3,968	63.9	3,964	60.9
Secondary	598	18.5	1,281	20.6	1611	24.7
Higher	76	2.3	305	4.9	355	5.45
*Place of residence*						
Urban	563	17.4	1,607	25.9	1,504	23.1
Rural	2,678	82.6	4,605	74.1	5,008	76.9
*Listens to radio*						
Never	89	2.7	552	8.8	495	7.6
Occasionally	317	9.8	741	11.9	782	12.0
Frequently	2,835	87.5	4,919	79.3	5,235	80.4
*Watches television*						
Never	1,393	43.0	2,779	44.7	2,251	34.6
Occasionally	1,044	32.2	1,557	25.1	2,323	35.7
Frequently	804	24.8	1,876	30.2	1,938	29.8
*Age at first sex (years*)						
Never	877	27.1	1,584	25.5	1,777	27.3
5–14	195	6.0	349	5.6	314	4.8
15–24	1,752	54.1	3,315	53.4	3,316	50.9
> 24	417	12.9	964	15.5	1,105	17.0
*Ever tested for HIV*						
No	914	28.2	1,186	19.1	2,234	34.3
Yes	2,327	71.8	5,026	80.9	4,278	65.7
*Wealth category*						
Middle	646	19.9	1,178	19.0	1,330	20.4
Poor	1,033	31.9	1,965	31.6	2,277	35.0
Rich	1,562	48.2	3,069	49.4	2,905	44.6

Source: Rwanda demographic and health surveys (2010–2020)

### Trends in the prevalence of male circumcision in Rwanda, 2010–2020

**Fig. 1:**
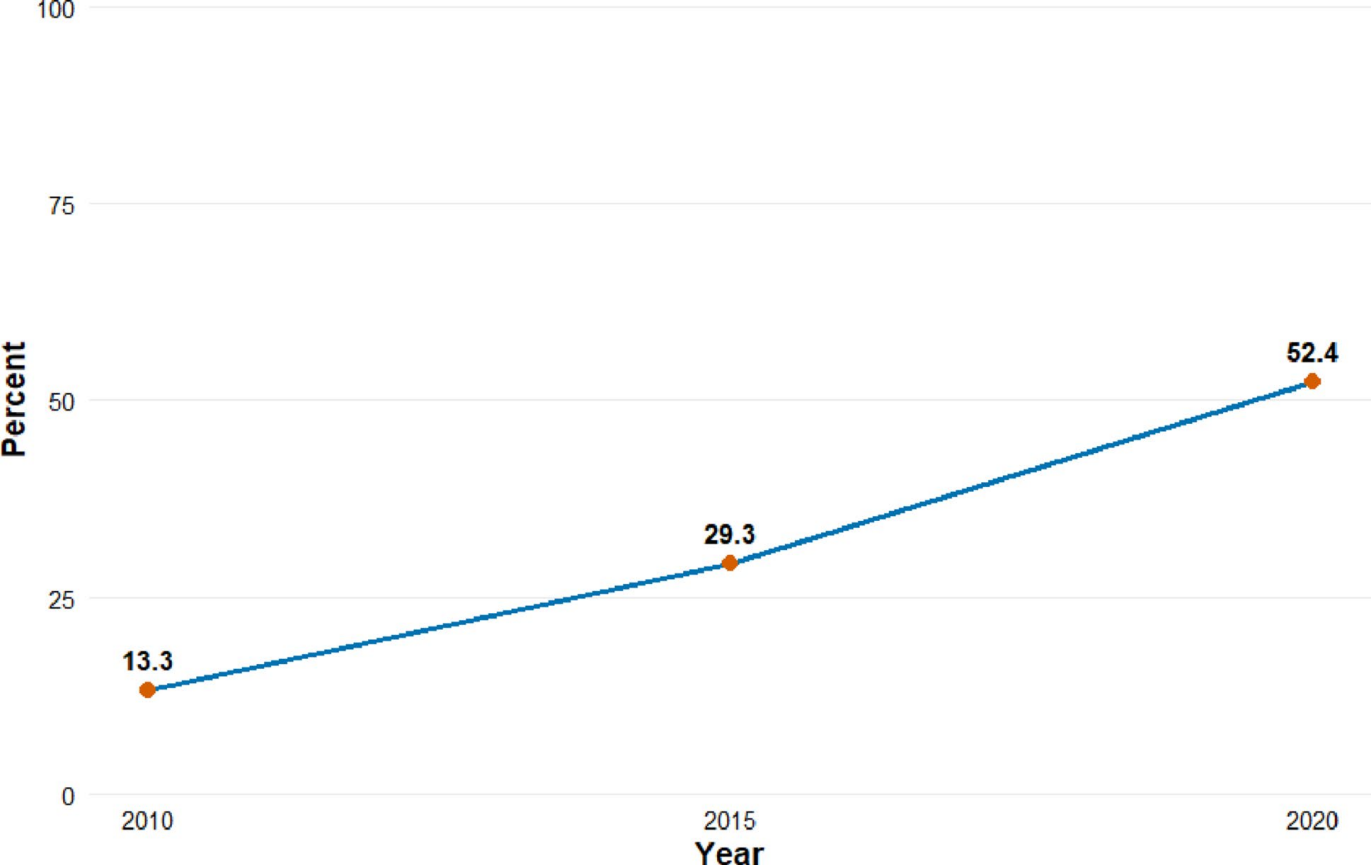
Trends in the Prevalence of Male Circumcision in Rwanda, 2010–2020

Figure 1 illustrates a notable upward trend in the prevalence of male circumcision in Rwanda from 2010 to 2020. In 2010, only 13.3% of men reported being circumcised. This figure more than doubled to 29.3% by 2015 and continued to rise significantly, reaching 52.4% in 2020. This substantial increase may reflect the impact of national health interventions and HIV prevention strategies that promote male circumcision as a public health measure, particularly given its recognized role in reducing the risk of HIV transmission.

### Kaplan–Meier analysis of time to male circumcision among Rwandan males

**Fig. 2:**
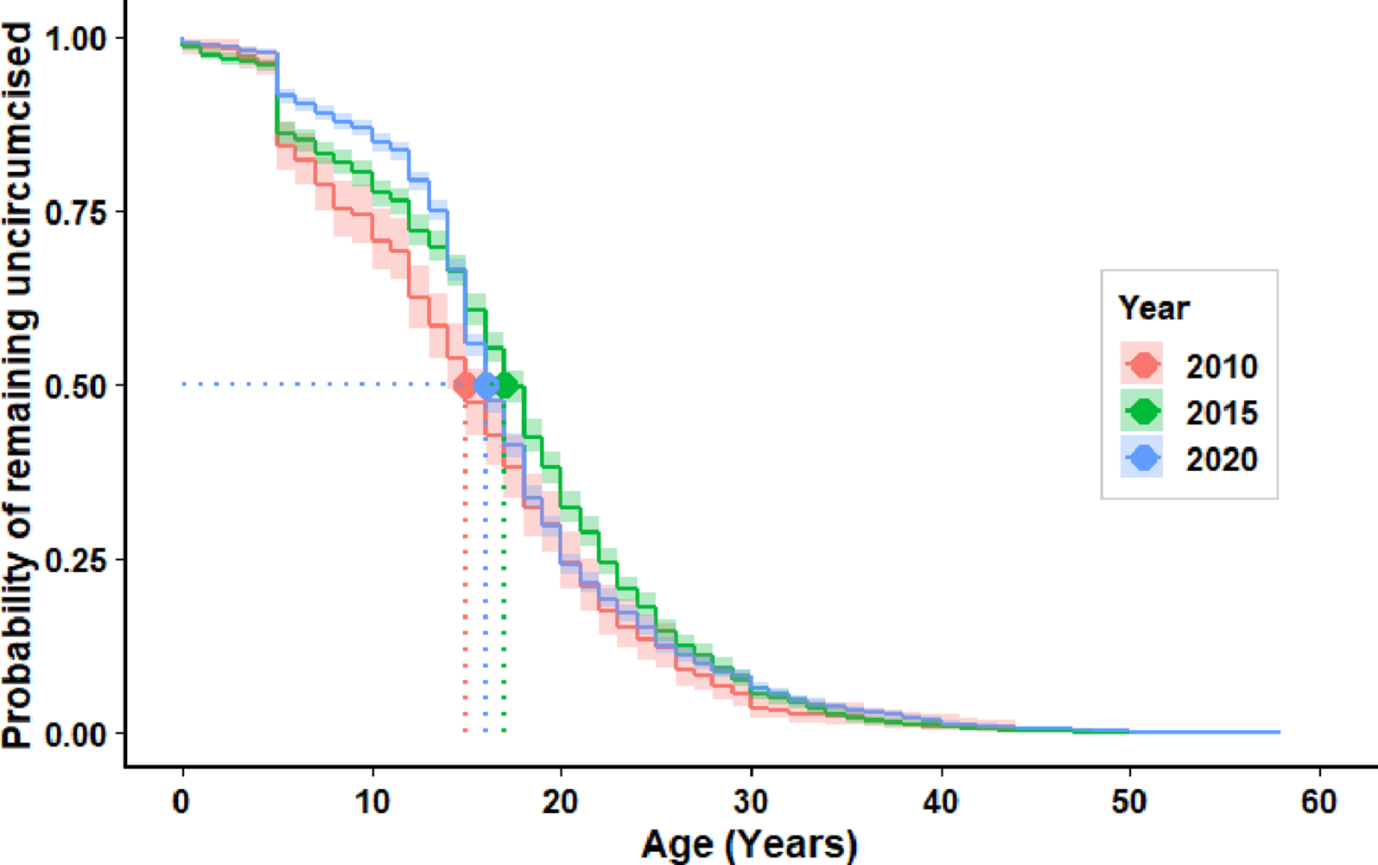
KM-Curve: Time to male circumcision in Rwanda

The median age at male circumcision showed notable variation across survey years, rising from 15 years in 2010 to 17 years in 2015 before declining to 16 years in 2020. This pattern indicates an initial slowdown in program expansion as efforts shifted to older males, followed by renewed progress in reaching younger age groups (Fig. 2).

### Comparison of median age at circumcision across socio-demographic groups: Rwanda DHS 2010–2020

In all three survey years, younger age groups consistently reported lower median ages at circumcision compared to older groups. The respondents aged 15–19 years, the median circumcision age remained fairly stable around 13–14 years across 2010, 2015, and 2020, while older groups such as 45–49 years and above 50 years had higher and more variable median ages, reaching up to 24 years in 2020.

Marital status also influenced trends, with single men having consistently younger median circumcision ages (14, 17, and 15 years) compared to married or separated men, whose medians rose over time. Education level showed a clear pattern: those with secondary education had lower median circumcision ages than those with primary or no education, with the secondary group showing an increase from 13 years in 2010 to 17 years in 2020.

Urban residents consistently underwent circumcision at younger ages than rural residents across the three surveys, although the gap narrowed slightly by 2020.

Media exposure had an effect with individuals who watched television frequently or listened to the radio frequently tended to have slightly lower median ages compared to those with limited or no media access, with minor fluctuations over time. Those who reported having their first sex after 24 years consistently had the highest median circumcision ages across surveys, increasing from 20 years in 2010 to 23 years in 2020.

Lastly, individuals who had ever been tested for HIV generally had higher circumcision ages than those who had never been tested, though the difference was relatively small, and wealth index differences across poor, middle, and rich categories were minor and remained relatively stable across years (Table 2).

**Table 2:** Median age at male circumcision by socio-demographic characteristics, Rwanda DHS 2010, 2015, and 2020

Variable	2010 Median age [95%CI]	2010 *p*-value	2015 Median age [95%CI]	2015 *p*-value	2020 Median age [95%CI]	2020 *p*-value
*Age Group (Years)*		< 0.001		< 0.001		< 0.001
15–19	13 [2.83–18.00]		14 [1.00–19.00]		14 [3.65–18.00]	
20–24	16 [3.18–22.80]		18 [1.00–23.00]		17 [5.00–22.00]	
25–29	18 [3.65–27.70]		21 [4.00–28.00]		19 [5.00–26.00]	
30–34	16 [3.40–30.00]		22 [1.00–33.00]		20 [5.00–32.00]	
35–39	13 [4.93–32.40]		18 [0.30–36.00]		24 [5.00–36.96]	
40–44	16 [5.00–39.60]		18 [3.80–40.00]		24 [5.00–41.00]	
45–49	14 [4.10–34.90]		21 [5.00–45.00]		23 [3.80 - 45.20]	
Above 50	17.5 [3.45–44.8]		16 [5.00–43.00]		18 [5.00–50.00]	
*Marital status*		< 0.001				< 0.001
Single	14 [3.25–28.00]		17 [1.00–29.00]		15 [4.00–24.42]	
Married	16 [3.00–36.90]		19 [2.30–39.00]		20 [5.00–42.73]	< 0.001
Separated	18.5 [6.12–37.60]		17[1.65–34.60]		18 [5.00–34.60]	
*Education Level*		< 0.001		< 0.001		< 0.001
No education	17 [0.00–44.40]		16[3.90 - 33.70]			
Primary	16 [5.00–32.10]		18 [2.00–37.00]		18 [5.00–43.00]	
Secondary	13 [3.00–30.00]		16 [1.00–30.00]		17 [5.00–40.00]	
Higher	16 [3.40–28.30]		19 [2.83–34.00]		15 [3.00–28.00]	
*Place of residence*		0.008		0.006	18 [5.00–32.00]	0.008
Urban	12.5 [3.00–30.00]		17 [1.00–34.00]		15 [1.00–38.00]	
Rural	16 [4.55–36.40]		18 [5.00–34.90]		16 [5.00–37.00]	
*Listen to Radio*		0.03		0.7		0.3
Never	13 [3.68–16.80]		17 [1.00–36.60]		16 [1.15 - 36.80]	
Occasionally	14 [0.00–26.60]		17 [1.00–35.10]		16 [5.00–40.00]	
Frequently	15 [4.00–34.40]		18 [2.00–34.0]		16 [5.00–37.10]	
*Watches television*		< 0.001		0.01		< 0.001
Never	18 [0.00–36.80]		18 [1.00–37.00]		17 [5.00–39.10]	
Occasionally	16.5 [5.00–36.80]		17 [4.13–34.00]		16 [5.00–37.00]	
Frequently	14 [3.73–30.00]		17 [2.00–34.00]		16 [3.00–35.00]	
*Age at first sex*		< 0.001		< 0.001		< 0.001
< 5 years	13 [4.13–23.80]		16 [1.00–25.00]		15 [4.00–21.30]	
5–14 years	12 [3.00–32.20]		15 [2.00–29.00]		15 [5.00–27.20]	
15–24 years	16 [3.00–32.90]		18 [2.00–35.00]		18 [5.00–40.00]	
> 24 years	20 [5.00–38.00]		22 [2.50 - 39.80]		23 [5.00–42.40]	
*Ever tested for HIV*		< 0.000		< 0.001		< 0.001
No	13 [2.03–30.00]		16 [1.00–26.00]		15 [5.00–34.60]	
Yes	16 [4.00–32.80]		18 [2.00–35.00]		17 [5.00–38.00]	
*Wealth index*		0.100		0.303		0.112
Poor	15.5 [4.35–38.60]		17 [3.00–34.00]		16 [5.00–32.00]	
Middle	15 [1.38–35.40]		18 [5.00–35.90]		16 [5.00–38.00]	
Rich	15 [3.00–30.70]		17 [1.00–35.00]		16 [3.00–38.00]	

Source: Rwanda demographic and health surveys (2010–2020)

### Determinants of early male circumcision among Rwandan men across DHS surveys (2010–2020)

The adjusted hazard ratios (AHRs) revealed distinct age-related differences in the timing of male circumcision across survey years (Table 3). Compared with men aged 15–19 years (reference group), older age groups consistently demonstrated lower hazards of circumcision, indicating that they tended to undergo circumcision at later ages. In 2010, the hazard of circumcision was 44% lower among men aged 20–24 years (AHR = 0.56; 95% CI: 0.39–0.80; *p* = 0.001) and 61% lower among those aged 25–29 years (AHR = 0.39; 95% CI: 0.26–0.59; *p* < 0.001). Similar trends were observed in 2015 and 2020, with the hazard decreasing progressively with age. By 2020, men aged 45–49 years had an AHR of 0.07 (95% CI: 0.05–0.09; *p* < 0.001), reflecting a 93% lower hazard of circumcision relative to those aged 15–19 years.

Educational attainment was positively associated with earlier circumcision. In 2020, men with higher education exhibited a 30% higher hazard of circumcision compared with those with no formal education (AHR = 1.30; 95% CI: 1.04–1.63; *p* = 0.02). Place of residence also influenced timing, with rural men showing persistently lower hazards of circumcision than urban men—AHRs of 0.81 (95% CI: 0.72–0.90; *p* < 0.001) in 2015 and 0.79 (95% CI: 0.72–0.85; *p* < 0.001) in 2020—indicating slower uptake in rural settings.

Media exposure played a substantial role. Frequent television viewing was associated with a higher hazard of circumcision across all survey years (2010 AHR = 1.53; 95% CI: 1.13–2.06; *p* = 0.01; 2015 AHR = 1.17; 95% CI: 1.02–1.33; *p* = 0.03; 2020 AHR = 1.21; 95% CI: 1.10–1.33; *p* < 0.001), suggesting that consistent access to media information accelerated circumcision uptake. Conversely, frequent radio listening was linked to lower hazards of circumcision in 2010 (AHR = 0.27; 95% CI: 0.10–0.78; *p* = 0.02) and 2020 (AHR = 0.81; 95% CI: 0.69–0.94; *p* = 0.01), indicating less timely uptake among regular radio listeners.

Sexual behavior also influenced timing. Men who reported sexual debut after age 24 had lower hazards of circumcision in 2015 (AHR = 0.73; 95% CI: 0.60–0.89; *p* < 0.001) and 2020 (AHR = 0.82; 95% CI: 0.70–0.95; *p* = 0.01), meaning circumcision occurred later among those initiating sex at older ages. In 2020, men who had ever tested for HIV had a slightly lower hazard of circumcision (AHR = 0.89; 95% CI: 0.82–0.96; *p* < 0.001), suggesting circumcision tended to occur later among those already engaged with HIV testing services. Additionally, in 2015, men from middle-income households exhibited lower hazards of circumcision compared with those from poorer households (AHR = 0.81; 95% CI: 0.68–0.96; *p* = 0.02), reflecting slower adoption among the middle-income group.

**Table 3:** Adjusted hazard ratios for factors associated with early male Circumcision, Rwanda DHS 2010–2020

Variable	2010 AHR (95% CI)	2010 *P*-value	2015 AHR [95% CI]	2015 *P*-value	2020 AHR [95% CI]	2020 *P*-value
*Age Group*						
15–19 years	ref		ref		ref	
Age 20–24	0.56 [0.39–0.80]	0.001	0.52 [0.45–0.61]	< 0.001	0.41 [0.36–0.46]	< 0.001
Age 25–29	0.39 [0.26–0.59]	< 0.001	0.33 [0.27–0.40]	< 0.001	0.24 [0.20–0.28]	< 0.001
Age 30–34	0.32 [0.20–0.51]	< 0.001	0.23 [0.19–0.29]	< 0.001	0.16 [0.14–0.20]	< 0.001
Age 35–39	0.39 [0.23–0.65]	< 0.001	0.21 [0.16–0.28]	< 0.001	0.10 [0.08–0.13]	< 0.001
Age 40–44	0.25 [0.14–0.44]	< 0.001	0.18 [0.13–0.24]	< 0.001	0.08 [0.06–0.10]	< 0.001
Age 45–49	0.34 [0.19–0.61]	< 0.001	0.14 [0.10–0.20]	< 0.001	0.07 [0.05–0.09]	< 0.001
Age Above 50	0.15 [0.08–0.28]	< 0.001	0.30 [0.22–0.41]	< 0.001	0.06 [0.05–0.08]	< 0.001
*Marital status*						
Single	ref		ref		ref	
Married	1.30 [0.97–1.73]	0.07	1.14 [0.98–1.33]	0.09	0.97 [0.86–1.11]	0.67
Separated	0.85 [0.39–1.83]	0.70	1.28 [0.89–1.85]	0.18	1.16 [0.88–1.54]	0.30
*Education level*						
No education	ref		ref		ref	
Primary	1.00 [0.65–1.53]	0.99	0.83 [0.64–1.07]	0.15	0.84 [0.70–1.01]	0.06
Secondary	1.25 [0.79–1.99]	0.31	1.01 [0.77–1.33]	0.92	1.10 [0.91–1.33]	0.33
Higher	1.15 [0.66–2.01]	0.60	1.00 [0.75–1.34]	0.99	1.30 [1.04–1.63]	0.02
*Place of residence*						
Urban	ref		ref		ref	
Rural	0.68 [0.53–0.87]	0.86	0.81 [0.72–0.90]	< 0.001	0.79 [0.72–0.85]	< 0.001
*Listen to Radio*						
Never	ref		ref		ref	
Less than once a week	0.44 [0.15–1.33]	0.15	0.95 [0.72–1.25]	0.79	0.85 [0.71–1.02]	0.08
At least once a week	0.27 [0.10–0.78]	0.02	0.94 [0.75–1.19]	0.74	0.81 [0.69–0.94]	0.01
*Watches television*						
Never	ref		ref		ref	
Less than once a week	1.30 [0.96–1.77]	0.11	1.20 [1.04–1.38]	0.01	1.08 [0.99–1.19]	< 0.001
At least once a week	1.53 [1.13–2.06]	0.01	1.17 [1.02–1.33]	0.03	1.21 [1.10–1.33]	< 0.001
*Age at first sex*						
< 5 years	ref		ref		ref	
5–14 years	0.92 [0.58–1.48]	0.75	1.01 [0.81–1.25]	0.95	0.93 [0.80–1.09]	0.38
15–24 years	0.88 [0.63–1.22]	0.43	0.86 [0.75–0.98]	0.03	0.94 [0.84–1.05]	0.25
Above 24 years	0.59 [0.37–0.92]	0.02	0.73 [0.60–0.89]	< 0.001	0.82 [0.70–0.95]	0.01
*Ever tested for HIV*						
No	ref		ref		ref	
Yes	0.84 [0.64–1.11]	0.23	0.89 [0.77–1.03]	0.12	0.89 [0.82–0.96]	< 0.001
*Wealth index*						
Poor	ref		ref		ref	
Middle	1.01 [0.68–1.48]	0.98	0.81 [0.68–0.96]	0.016628	0.95 [0.85–1.06]	0.34
Rich	0.88 [0.64–1.23]	0.47	0.87 [0.75–1.02]	0.073743	1.02 [0.93–1.13]	0.62

Ref: reference category

Source: Rwanda demographic and health surveys (2010–2020)

## Discussion

This study demonstrated a significant increase in the prevalence of male circumcision in Rwanda from 13.3% in 2010 to 52.4% in 2020, accompanied by a trend toward younger ages at circumcision. These findings align with the global and regional efforts promoting early male circumcision as a public health intervention for HIV prevention and other health benefits [17]. 

The present analysis aligns with earlier evidence demonstrating Rwanda’s substantial progress in expanding voluntary medical male circumcision (VMMC) services [18]. reported that 69% of Rwanda Defense Force (RDF) members were circumcised, with 44% of procedures occurring within the two years preceding data collection, reflecting rapid scale-up within a structured institutional setting. In contrast, our population-based findings from the Rwanda Demographic and Health Surveys (2010–2020) show a steady national increase from 13.3% in 2010 to 52.4% in 2020, with the median age at circumcision declining from 17 years in 2015 to 16 years in 2020.

When comparing the age trends, our findings are consistent with the results from Mozambique, where scaling up circumcisions among adolescents and young adults, particularly those aged 15–24 years, was found to have the most substantial long-term impact on reducing HIV incidence [19]. In our study, men aged 20–24 and older were significantly less likely to undergo early circumcision compared to those aged 15–19 years, highlighting the critical window of opportunity during adolescence for scaling up male circumcision efforts.

The influence of education observed in our study, where higher education was associated with increased likelihood of early circumcision in 2020, mirrors the findings from Ethiopia [20], where knowledge and positive attitudes significantly predicted infant circumcision practices. This underscores the role of health education and awareness in promoting circumcision uptake.

In addition, we found that media exposure, particularly frequent television watching, was associated with higher likelihood of early circumcision. This aligns with findings from another study conducted in Kigali, where community encouragement and access to circumcision information were significantly associated with positive attitudes and knowledge toward circumcision [21]. Mass media campaigns may therefore play an essential role in shaping social norms and influencing decision-making around circumcision.

Regionally [8], showed that male circumcision prevalence in sub-Saharan Africa rose from 40.2% during 2010–2015 to 56.2% in 2016–2023, with particularly higher rates in Eastern Africa, including Rwanda. Our observed increase in circumcision prevalence in Rwanda matches these broader continental trends, suggesting the success of national and regional efforts aligned with WHO and UNAIDS targets.

The findings from this study reaffirm that younger age, urban residence, higher education, and exposure to media are key drivers of early male circumcision uptake [12]. They highlight the need for tailored interventions focusing on adolescents, rural populations, and less-educated groups to accelerate circumcision coverage and achieve public health goals.

## Conclusions

This study revealed a significant increase in the prevalence of male circumcision in Rwanda between 2010 and 2020, with a notable shift toward earlier circumcision ages. Several factors, including younger age, higher education, urban residence, frequent media exposure, and HIV testing history, were significantly associated with early circumcision. The findings emphasize the importance of strengthening interventions targeting adolescents and young adults, especially in rural and less-educated populations, to further improve circumcision coverage and contribute to HIV prevention efforts. Programs promoting early circumcision should integrate educational campaigns and culturally sensitive strategies to optimize uptake.

## Data Availability

The datasets analysed during the current study are publicly available from the DHS Program website ([https://dhsprogram.com/](https:/dhsprogram.com)) upon request.

## Abbreviations

AHR: Adjusted Hazard Ratio
CI: Confidence Interval
DHS: Demographic and Health Survey
GovRw: Government of Rwanda
HIV: Human Immunodeficiency Virus
MC: Male Circumcision
RDF: Rwanda Defense Force
UNAIDS: Joint United Nations Programme on HIV/AIDS
VMMC: Voluntary Medical Male Circumcision
WHO: World Health Organization
